# Myxedematous Coma

**Published:** 1911-10

**Authors:** 


### Myxedematous Coma

HERTOGHE, *Acad. de Belgique,* February 25, 1911, states that this occurs late in the disease, and is due to some exciting cause, dietetic error, infection, intercurrent intoxication, and the like. It may follow the lines of diabetic or uremic coma (Are these really different?) the differential diagnosis being difficult if there is a complicating essential albuminuria. He considers that the coma is both toxemic and mechanic, since the brain is subject to the same infiltration as other tissues, whence compression symptoms ensue.

